# Editorial: Ethical and sustainable food choice: drivers and health effects

**DOI:** 10.3389/fnut.2024.1455664

**Published:** 2024-07-25

**Authors:** Gianluca Rizzo, Maximilian Andreas Storz

**Affiliations:** ^1^Independent Researcher, Messina, Italy; ^2^Center for Complementary Medicine, Department of Medicine II, Faculty of Medicine, Medical Center, University of Freiburg, Freiburg, Germany

**Keywords:** vegetarian, vegan, plant-based, flexitarian, climate change, environmental, ethical, sustainability

Since the lack of fair and appropriate access to food is a global public health problem affecting many populations, a transition toward a more sustainable food system is urgently required (1). While in low-income countries food access is often limited due to disadvantageous socio-economic conditions, high-income countries are nowadays frequently characterized by an excess supply of low-quality, energy-dense foods. The use of highly processed foodstuffs and poor dietary habits favor the onset of non-communicable chronic diseases, which could be largely prevented by adopting a plant-based diet (2, 3). At the same time, countries with a rapid urbanization trend are adopting an increasingly Westernized diet with a prevalence of meat-based food options which poses strong health risks and an ecological impact that is not sustainable (4, 5).

If we consider social, economic, health, and environmental aspects, a global system that ensures fair and regular access to high quality foods must include a broad concept of sustainability that embraces all these aspects (6, 7). In particular, the typical diet of high-income countries, based on a large use of foods of animal origin, requires greater resources starting from the use of agriculture intended for the production of feed for livestock, with a more expensive environmental cost and a greater impact from by-products such as greenhouse gases (8). Arguably, a transition toward a diet based mostly on plant foods can satisfy all social, health and environmental critical issues, guaranteeing sustainable agriculture and promoting more homogeneous access to food among low-income countries and industrialized ones (9, 10).

In this context, the drivers and barriers to encourage the adoption of more sustainable food choices cannot be underestimated (11). Dietary modifications frequently involve resistance by individuals and a correct and sensitive communication of the possible advantages for the planet and human health could have favorable effects (12).

In the pilot study “*A multi-center prospective study of plant-based nutritional support in adult community-based patients at risk of disease-related malnutrition*,” Delsoglio et al. enrolled 24 adults at risk of malnutrition who required nutritional support. The study aimed to verify the acceptability of a vegan option of an oral nutritional supplement. Taking into account the increase in demand for plant-based options, the authors studied the acceptability of a particular formulation that could satisfy this type of request. Participants were followed for 28 days during which they received dietary advice and support. The results suggested excellent compliance and tolerance with maintenance of appetite. While the nutritional status of participants improved subsequent to a higher energy intake (mainly from protein), malnutrition risk decreased.

The impact of food choices on health and the environment was explored in the manuscript “*Identification of three dietary groups in French university students and their associations with nutritional quality and environmental impact*” Arrazat et al., examined a representative sample of 582 French students. Based on data from a 125-item food frequency questionnaire, the three distinct dietary patterns emerged. While 20% of the participants adopted a diet with high health indices but a high impact on the environment through greenhouse gas emissions, the other participants were equally divided between a Westernized diet, characterized by a high environmental impact and low health indices, and a frugal diet with lower environmental impact and intermediate health indices. These results highlight that among some segments of the population, it may still be necessary to adopt a dietary style which combines health and environmental advantages. The social determinants that emerged from this study (economic availability, age, and cohabitation in the family of origin) could help in this task.

Consistently, Patel et al. in “*Testing the effect of descriptive dynamic social norm messages on meatless food purchases in Aotearoa New Zealand and UK university food outlets*” conducted 2 parallel week-long intervention studies (in New Zealand and the UK) to test the effectiveness of social norm messages to reduce the consumption of animal-based foods. However, messages displayed in university food outlets and via social media did not appear to be effective in changing meat and meatless food purchases. This suggests that modifying eating behavior is challenging, and complex mechanisms may be taken into account. An approach not limited to descriptive dynamic social norm interventions may be warranted. Customers at food outlets could be profoundly influenced by social aspects that stimulate the maintenance of meat-based eating behaviors while at the same time, they could ignore messages that discourage a choice that they may have already planned at that moment. However, it should be mentioned that awareness of the messages was low in both studies, as emerged from a survey conducted among a subgroup of customers.

Resistance to the transition to a plant-based diet often stems from a misinterpretation of the possible risks associated with adopting a vegetarian diet. This may apply in particular to bone health, which is controversially discussed (13). However, as highlighted by Galchenko et al. in the cross-sectional study entitled “*Bone mineral density parameters and related nutritional factors in vegans, lacto-ovo-vegetarians, and omnivores: a cross-sectional study*,” there appeared no difference in bone mineral density among three different dietary patterns. Forty-four omnivores, 38 lacto-ovo vegetarians, and 46 vegans from Russia consumed less vitamin D than recommended without significant differences among groups. Vegans showed higher levels of PTH than omnivores but still within the normal range. This suggests that it is not the dietary lifestyle itself that represents possible deficiencies but some nutrients deserve particular attention when planning a sustainable diet.

Among the foods that can guide the dietary transition toward greater sustainability, legumes and common beans emerge as promising options, according to the review “*Legumes and common beans in sustainable diets: nutritional quality, environmental benefits, spread and use in food preparations*,” Lisciani et al. showed that the environmental and health sustainability, cost-effectiveness and versatility of legumes and common beans in traditional recipes and second-generation foods could easily guarantee fair access to food in low-income countries while mitigating the food excesses of industrialized countries.

Overall, this Research Topic brings together contributions emphasizing the importance of providing plant-based alternatives that can satisfy the growing demand among the world population but at the same time the need to implement effective strategies for a more sustainable food transition.

## Author contributions

GR: Writing – original draft. MS: Writing – review & editing.
